# Filarial Periodicity

**Published:** 1899-09-16

**Authors:** 


### FILARIAL PERIODICITY.

Dr. Patrick Manson, speaking at Portsmouth, set himself four questions concerning that curious phenomenon—filarial periodicity—the fact that in the blood of those suffering from filaria disease the embryos appear in countless swarms in the cutaneous circulation during the night, and disappear from it during the day: (1) What is the object of this? (2) Is it constant? (3) What becomes of the young filariæ during the day? (4) If they retire during the day to some internal organ, how is this brought about?

1. As to the object, it is now known that the presence of the embryos in the cutaneous circulation occurs in correspondence with the nocturnal appearance of the mosquito, which is an efficient intermediary host for the filaria.

2. This periodicity is constant except during fever, or when the habits in regard to the time of sleeping and waking are inverted. In the latter case the periodicity is also inverted.

3. As to what becomes of the filaria during the day, this question Dr. Manson is now able to answer. He had already ascertained that the periodicity was not due to the life of the embryo being short and to a fresh swarm being discharged by the parent worm every evening. The question remained, Where did they hide themselves during the day? This was answered by an examination of the body of a man whose blood was known to contain Filaria nocturna, and who committed suicide by means of prussic acid at half-past eight a.m., that is, just about the time when the embryos would have retired from the cutaneous circulation. The result of this examination was to show that during its temporary absence from the cutaneous circulation the Filaria nocturna is present in the larger blood vessels, particularly the arteries; that a few are to be found in the capillaries of the muscles and brain, a few in the vessels of the kidneys, a considerable number in the muscles of the heart; but that the majority are lodged in the blood vessels of the lungs, occurring in clusters in the larger vessels, or singly and more or less coiled up in the capillaries.

4. As to the mechanism by which this alternation of localisation by day and by night is effected nothing is known.

